# Impact strength of denture base and reline acrylic resins: An in vitro study

**DOI:** 10.1177/1758736012459535

**Published:** 2012-09-12

**Authors:** Ana L Machado, Bruna C Bochio, Amanda F Wady, Janaina H Jorge, Sebastião V Canevarolo, Carlos E Vergani

**Affiliations:** 1 Department of Dental Materials and Prosthodontics, Araraquara Dental School, UNESP—Univ Estadual Paulista, Araraquara, SP, Brazil; 2 Department of Materials Engineering, São Carlos Federal University, São Carlos, SP, Brazil

**Keywords:** Impact strength, denture base, acrylic resins

## Abstract

This study evaluated the impact strength of a denture base resin (Lucitone 550—L) and four reline resins (Tokuyama Rebase II—T; Ufi Gel Hard—U; New Truliner—NT, and Kooliner—K), both intact and in a reline combination (L/L, L/T, L/U, L/NT, and L/K). For each group (n = 20), half of the specimens were thermocycled before testing. Charpy tests were performed, and the impact strengths were calculated. Data were analyzed by two-way analyses of variance and Tukey’s test (p = 0.05). For the intact groups, mean impact strength values for L (1.65 and 1.50) were significantly higher than those of the reline resins (0.38–1.17). For the relined groups, the highest mean impact strength values were produced by L/T (5.76 and 5.12), L/NT (6.20 and 6.03), and L/K (5.60 and 5.31) and the lowest by L/U (0.76 and 0.78). There were no significant differences between L and L/L. Thermocycling reduced the impact strength of T (from 0.73 to 0.38) and L/L (from 1.82 to 1.56).

## Introduction

The proper functioning of hard chairside reline resins depends to a great extent on their characteristics and mechanical properties. Therefore, previous studies of autopolymerizing reline resins have examined their chemical compositions and residual monomer content^1,2^; physical and mechanical properties such as hardness,^3,4^ flexural strength,^4,5^ water sorption and solubility,^1^ and porosity^6,7^; and bond strength to the heat-polymerized denture base resins.^8–11^ Impact strength (IS) is also a desirable property because it is a measure of the energy required to initiate and propagate a crack through the material. Thus, it can reflect the contact force needed to cause fracture in a denture under situations such as accidental dropping.^12^ The occurrence of fracture, observed in maxillary and mandibular removable prostheses,^13^ results in additional costs, as well as discomfort to patients^14^ as they must be without the dentures during the laboratory procedures required to repair or replace the broken denture.

Because dentures are exposed to temperature changes as a result of ambient temperature changes and the intake of hot/cold foods,^15^ resistibility to thermal stresses is also another important consideration clinically. Therefore, studies have used thermal cycling to simulate the clinical conditions of the oral cavity.^5,11,16^ The presence of an interface between the denture base resin/relining material may also have an influence on the magnitude of the effects resulting from the changes in temperature. The bond between reline materials and denture base resins is obtained by interpenetration and interpenetrating polymer network formation at the interface.^10,17^ Archadian et al.^5^ evaluated different combinations between denture base and reline resins and found that some of the polymer combinations showed a decrease in the flexural strength after thermocycling, demonstrating that the thermal effect caused weakening of the bond between the materials. Besides, some adhesive failures were observed. Similar results were obtained in the study by Minami et al.,^11^ which investigated the bond strength between a high-impact denture base resin and two autopolymerizing resins, one used as a denture repair material and the other marketed as a relining material. The bond strength of the reline resin was significantly lower than that of the repair resin, under all conditions evaluated. In addition, the bond strength of the reline resin significantly decreased after thermal cycling, regardless of the conditions of surface treatment and water content. These findings were attributed to the high-molecular-weight monomers used in the reline resins, which may not penetrate into the polymer structure of the denture base resin, thus resulting in a poorly interpenetrating network.^5,11^ In addition, if there is a difference in the integration between the reline and the denture base polymers, microcracks are likely to occur at their interface after thermocycling.^5^ On the other hand, the results from Azevedo et al.^18^ indicated that for some relining materials, the bond strength could increase after storage in water at 37°C, suggesting a continued polymerization reaction of both the interpenetrating polymer formed at the interfacial region and the relining material located close to the interface.

A review of the available literature reveals that, to date, little information is available on the IS of autopolymerizing reline materials.^19–21^ Moreover, the effect of thermal stresses on the IS of denture base and reline resins has not been investigated. Thus, the aim of this study was to evaluate the IS of one denture base acrylic resin and four hard chairside reline resins, tested alone (bulk specimens) or in combination (denture base/reline materials). The effect of thermocycling on the IS of bulk and relined specimens of each resin was also evaluated. The null hypothesis was that IS was not determined by the (a) choice of intact material, (b) presence of a reline, and (c) thermocycling, within the parameters of the experiment.

## Material and methods

One heat-polymerized denture base acrylic resin and four autopolymerizing reline resins were selected for this study (Table 1). Initially, rectangular bars (60 × 6 × 2 mm) from Lucitone 550 (L) material were prepared. The material was proportioned, and after the mixture reached the dough stage, it was inserted into the mold in a dental flask and packed. Molds were prepared by investing silicone patterns (Zetaplus Putty; Zhermack, Rovigo, Italy), placed between two glass slides, in type IV stone (Troquel Quatro; Polidental Manufacturing and Trade Ltd, SP, Brazil). The denture base acrylic resin was polymerized in a thermostatically controlled water bath (Termotron P-100; Termotron, Piracicaba, SP, Brazil) according to the manufacturer’s recommendations (Table 1). After polymerization, the flasks were left on the bench to cool at room temperature for 30 min and then placed for 15 min under running water before opening, following the manufacturer’s instructions. Thereafter, the L rectangular bars were removed from the flasks, the edges were finished with 400-grit silicon carbide paper, and the accuracy of the dimensions was verified at three locations for each dimension. A tolerance of ±0.03 mm was accepted. The L rectangular bars were stored in distilled water at 37°C ± 1°C for 50 ± 2 h before relining.^22^ After water storage, the denture base resin surfaces to be bonded with the reline materials were ground wet in an automatic grinding machine (Metaserv 2000, model 95-2829; Buehler UK Ltd, Coventry, England) using silicon carbide paper (240 grit).^18,20^ The surfaces were brushed with liquid detergent for 20 s, rinsed with distilled water, and dried with absorbent paper.

**Table 1.** Materials used in this study

Material	Code	Batch no.	Manufacturers	Composition		Powder/liquid ratio (g/mL)	Polymerization cycles
				Powder	Liquid		
Lucitone 550	L	P-303758 and L-213457	Dentsply Indústria e Comércio Ltda., Petrópolis, Rio de Janeiro, Brazil	PMMA	MMA and EDGMA	2.1/1.0	90 min at 73°C and 30 min at 100°C
Tokuyama Rebase II	T	UF64145	Tokuyama Dental Corp., Tokyo, Japan	PEMA	AAEM and 1,9-ND	2.4/1.0	5.5 min at room temperature
Ufi Gel Hard	U	631742	VOCO, Cuxhaven, Germany	PEMA	1,6-HDMA	2.12/1.2	7 min at room temperature
New Truliner	NT	0310528	The Bosworth Co., Skokie, IL, USA	PEMA	IBMA and DBP	1.34/1.0	20 min at room temperature
Kooliner	K	0508187	GC America Inc., Alsip, IL, USA	PEMA	IBMA	2.1/1.0	10 min at room temperature

PMMA: poly(methyl methacrylate); PEMA: poly(ethyl methacrylate); MMA: methyl methacrylate; EDGMA: ethylene glycol dimethacrylate; AAEM: 2-(acetoacetoxy) ethyl methacrylate; 1,9-ND: 1,9-nonanediol dimethacrylate; 1,6-HDMA: 1,6-hexanediol dimethacrylate; IBMA: isobutyl methacrylate; DBP: di-*n*-butyl phthalate.

Before the L rectangular bars are relined with the autopolymerizing materials New Truliner (NT), Tokuyama Rebase II (T), and Ufi Gel Hard (U), the bond surfaces were treated with the bonding agents recommended by the manufacturers, whereas for the reline resin Kooliner (K), methyl methacrylate (MMA) monomer was applied for 180 s.^9^

The relined specimens were fabricated using a stainless steel mold with a cavity of 60 × 6 × 4 mm. The L rectangular bars were placed into the cavity, and the reline resins were inserted to fill the remainder of the mold. The manufacturers’ instructions of each reline material for mixing and processing were followed (Table 1). An acetate sheet and a glass plate were placed over the reline material, and pressure was applied until polymerization was complete. The edges of the specimens were finished, and the accuracy of the dimensions (width and thickness) was verified with a caliper (Mitutoyo Sul Americana, Suzano, SP, Brazil) at three locations of each dimension to within 0.03 mm tolerance.^20^

To evaluate the IS of the specimens made following laboratory reline procedures, L rectangular bars were relined with the same material. Initially, silicone patterns (60 × 6 × 4 mm) were obtained, placed between two glass slides, and then flasked to create molds for packing the L–L specimens. The L rectangular bars (2.0 mm) were then adapted in the lower portion of the stone mold, and the remaining 2.0 mm was filled with L acrylic resin dough. Processing, finishing, and verification of the accuracy of the specimens were performed as described.

For comparison purposes, the bulk specimens of all materials were prepared to the thickness of the relined specimens (4 mm) and tested. Half of the bulk (n = 20) and half of the relined (n = 20) specimens of each material were thermocycled before testing. Thermal cycles were made in a thermocycling machine (model MSCT-3; Marcelo Nucci—ME, São Carlos, SP, Brazil) and consisted of 5000 cycles at 5°C and 55°C with a 30-s dwell time.^16^

For K, NT, T, and U reline resins, the specimens (bulk and relined) were subjected to the impact tests, or to the thermal cycling prior to the impact tests, within 30 min after polymerization. This time period was used as the patients will be wearing the relined denture bases soon after polymerization. For the L denture base material, the specimens (bulk and relined with the same material) were subjected to the impact tests, or to the thermal cycling prior to the impact tests, after storage in distilled water at 37°C ± 1°C for 50 ± 2 h.^22^

Before testing, the specimens were notched with a notching cutter (Notchvis; Ceast, Pianezza, Italy). The V-notches were cut into the reline materials, across the width of the specimens with a depth of 0.8 mm leaving an effective depth under the notch of 3.2 mm.^20^ The ISs were evaluated by the Charpy impact tester (RESIL 25R; Ceast, Pianezza, Italy), with the unnotched surface of the specimens facing the pendulum. The test was performed with 0.5-J pendulum and a 150° lifting angle. The IS (in kJ/m^2^) was calculated as IS = E_C_/(hb_A_), where E_C_ is the corrected energy absorbed by breaking the test specimen, b_A_ is the remaining thickness at the notch tip, and h is the width of the specimen.

For experimental conditions L/T, L/U, L/NT, and L/K, delamination between the denture base and reline resin was observed for the majority of the samples, and the results were distinct from those of the other conditions evaluated. Thus, it was considered appropriate to analyze their data separately, using a two-way analysis of variance (ANOVA) with the factors material and group. A separate two-way ANOVA was also used to test for differences among the other experimental conditions (L, T, U, NT, K, and L/L). Levene and Shapiro–Wilk tests (Statistica 6.0; Statsoft, Tulsa, OK, USA) were applied to test homogeneity of variance and normality, respectively. Tukey’s honestly significant difference (HSD) post hoc test was applied to the results to determine whether significant differences existed among the means. A significance level of p ≤ 0.05 was established.

## Results

The mean IS values and the standard deviations for all conditions evaluated are shown in Table 2. For the intact groups, the mean IS of L was higher than that of K, and both were higher than those of T, U, and NT. For the relined groups, the highest IS values were seen for L/T, L/NT, and L/K, and the lowest IS values were seen for L/U specimens. There were no significant differences between L bulk and L/L specimens.

**Table 2.** Impact strength mean values (kJ/m^2^) and standard deviations (SDs) of acrylic resins and groups evaluated

Materials	Groups	
	Nonthermocycled	Thermocycled
L	1.65 (0.14)^**Ca**^	1.50 (0.19)^**Ea**^
T	0.73 (0.06)^**Aa**^	0.38 (0.15)^**Ab**^
U	0.58 (0.11)^**Aa**^	0.67 (0.12)^**BCa**^
NT	0.61 (0.06)^**Aa**^	0.52 (0.08)^**ABa**^
K	1.17 (0.14)^**Ba**^	1.02 (0.06)^**Da**^
L/L	1.82 (0.27)^**Ca**^	1.56 (0.26)^**Eb**^
L/T	5.76 (1.33)^**Da**^	5.12 (1.82)^**Fa**^
L/U	0.76 (0.16)^**Aa**^	0.78 (0.16)^**Ca**^
L/NT	6.20 (1.74)^**Da**^	6.03 (2.23)^**Fa**^
L/K	5.60 (3.34)^**Da**^	5.31 (2.53)^**Fa**^

Horizontally, identical superscripted small letters denote no significant differences among groups (p > 0.05). Vertically, identical superscripted capital letters denote no significant differences among materials (p > 0.05).

Table 2 also shows that thermocycling reduced the IS of T, while the other intact materials (L, U, NT, and K) were not significantly affected. For the relined groups, the results demonstrated that L/L specimens exhibited a small but significant reduction in the IS after thermocycling. No significant differences were found between nonthermocycled and thermocycled specimens for the other reline combinations (L/T, L/U, L/NT, and L/K).

All specimens broken with a sharp fracture typical of the brittle fracture behavior, characterized by a lack of distortion of the broken parts. In addition, several specimens of the experimental conditions L/T, L/NT, and L/K showed delamination between the denture base resin and the relining material.

## Discussion

The null hypothesis that the IS was not determined by the (a) choice of intact material, (b) presence of a reline, and (c) thermocycling, within the parameters of the experiment, was rejected. It is clear that the mean values of IS for resin L were the highest of the intact materials tested. A study^2^ reported the residual monomer content of some denture base and reline resins. Resin L, when polymerized by a short cycle (which was also used in the present investigation), showed a lower percentage of residual monomer (0.08%) than the reline resins K (1.52%) and U (0.45%). The residual monomer content may have an adverse effect on mechanical properties such as hardness^4^ and tensile strength.^23^ However, a direct relationship between the quantity of residual monomer and IS could not be observed in the present study. The differences in composition of the materials evaluated could help explain, at least partly, their different behaviors when subjected to the impact test. The powder of resin L consists of polymethyl methacrylate (PMMA), while the liquid contains MMA monomer and the cross-linking agent ethylene glycol dimethacrylate (EDGMA). Although the manufacturer does not provide information on the exact concentration of EDGMA in the resin L, the percentage of this cross-linking agent in denture base acrylic resins usually is limited to 15%.^1,24^ For the reline resin K, the powder is essentially polyethyl methacrylate (PEMA), and the liquid contains isobutyl methacrylate (IBMA), without a cross-linking agent. The powder of resins U and T also consists of PEMA. However, the liquid of material U is mainly composed of the cross-linking agent 1,6-hexanediol dimethacrylate (1,6-HDMA). For material T, the liquid consists of 59% acetoacetoxy ethyl methacrylate (AAEM) monomer and 39% of 1,9-nonanediol dimethacrylate (1,9-ND) as the cross-linking agent. Cross-linking agent concentration influenced the IS of denture base acrylic resin,^25^ and an increase in the cross-linking reaction decreased the flowability of polymer, resulting in the reduction of the IS.^20,26^ Thus, the lower IS values of T and U compared to L and K resins could be related to their higher concentration of cross-linking agents. It is likely that during the polymerization reaction of T and U materials, a highly cross-linked network was formed, thus resulting in polymers with reduced IS. In spite of the composition of resin NT being similar to that of K, the IS value of the former was significantly lower than the latter. A possible explanation could be the presence of 8% plasticizer agent (di-*n*-butyl phthalate (DBP)) in the liquid of material NT,^1^ which may have an influence on its resistance to impact forces.

The mean values of IS for L/L were greater than the corresponding values for the intact material L, indicating that when relining is performed with the same heat-polymerized resin used for denture base fabrication, the IS of the denture is not detrimentally affected. A possible explanation for these results could be the bond between the two materials. Some researchers have looked at solvent bonding since it gives relatively strong bonding, without introducing a foreign adhesive material. The mechanism for solvent bonding is through the dissolution of the polymer from both bonding surfaces followed by the interdiffusion of polymer chains.^27,28^ Similar mechanism has been used for bonding in denture repair.^11,29–32^ In the relining procedure, the bond between resins is due to the diffusion and polymerization of monomer across the reline resins–denture base interface to form interpenetrating polymer networks.^10,17^ For interlacing, the solubility parameter of the solvent monomer should match the solubility parameter of the polymer.^33^ With the basic principle of “like dissolves like,” polymers will be soluble in liquids that have solubility parameters not too different from theirs.^27^ The solubility parameter of MMA (8.8 (cal/cm^3^)^1/2^) is similar to that of PMMA (9.45 (cal/cm^3^)^1/2^).^33^ In addition, the resin L remained under pressure for 30 min in contact with the polymerized denture base resin, before polymerization, and was processed at higher temperatures, with a terminal boil of 30 min. All these factors may have enhanced the diffusion of the monomer MMA, present in the dough mixture, from the reline material into the denture base PMMA polymer,^17,28^ promoting an adequate bond and strong interface between the materials. During the impact tests of L/L specimens, no delamination between the two materials was observed. This further suggests that the interfacial bonding was strong enough and that the cracks may have propagated in the relined specimens as if through a homogeneous material.^20^ As a result, no significant differences were found between L bulk intact specimens and L relined specimens.

As stated for the L/L specimens, the bond between the denture base L and the hard reline materials may have also contributed to the results of the present study. For the L/NT specimens, the L bond surfaces were treated with the adhesive supplied by the reline resin NT manufacturer, which contains MMA that is also the main constituent of the resin L liquid used for treating the bonding surfaces during the fabrication of the L/K specimens. Despite the similarity of the solubility parameters of MMA and PMMA, the contact time between the solvent and the bonding surfaces was much lower compared to that of the L/L specimens (30 min of dough time). In addition, the molecular weight of IBMA (142.19 g/mol),^34^ the main component of NT and K liquids, is higher than that of MMA (100.18 g/mol).^34^ Larger molecules may act slower with respect to swelling PMMA and penetrating the PMMA surface. For the L/T specimens, the L bond surfaces were treated with the adhesive supplied by the reline resin T manufacturer, which contains the nonpolymerizable solvents, ethyl acetate (EA) and acetone (A).^29^ Their solubility parameters are close to that of PMMA (EA: 9.1 (cal/cm^3^)^1/2^; A: 9.9 (cal/cm^3^)^1/2^).^33,35^ The surfaces to be bonded with the reline resin U were treated with the bonding agent recommended by the manufacturer, which contains a monomer (2-hydroxyethyl methacrylate (HEMA)) and a solvent (dichloromethane (DCH)), with solubility parameters of 11.4 and 9.68 (cal/cm^3^)^1/2^, respectively.^35^ Although the solubility parameter of HEMA is higher than that of PMMA, bonding agents containing both solvents and monomers have been reported to have a positive effect on the bond strength of denture base to reline resins,^10,18^ and the use of a DCH-based primer was more effective than the EA-based primer.^30^ The application of MMA and Tokuyama Rebase II adhesive on heat-polymerized acrylic resin resulted only in shallow pits and small crests, respectively, in contrast with the three-dimensional (3D) pores produced by other solvents.^29^ In addition, it has been found that the reline resin U exhibited higher mean shear bond strength values to the denture base acrylic resin L than other hard reliners,^36^ including K and NT materials.^18^ The molecular weight of the monomers may also have played a role. Reline resin U contains 1,6-HDMA with a molecular weight of 254.33 g/mol.^34^ For material T, although the molecular weight of the monomer AAEM is 214.22 g/mol,^34^ this reline resin also contains 39% of the cross-linking agent 1,9-ND, which has a higher molecular weight of 296.4 g/mol.^34^ Thus, it is likely that for the hard chairside reline T, NT, and K, the interfacial bonding with the denture base resin L was not as strong as the bond achieved when the relining material was the same as that of the denture base (L/L combinations) or when the specimens were relined with resin U (L/U).

During the tests, a large number of L/T, L/NT, and L/K specimens showed delamination between the reline resin and the denture base resin, while for L/U specimens, no delamination was observed. Studies on composites have shown that the propagation of fracture during loading initiates from microdamage of the polymer matrix and results in transverse matrix cracking, delamination, and finally to fiber failure.^37^ It has been observed that when the fiber-rich area was covered with the polymer, the fracture propagated until it reached the fibers and continued as delamination between the reinforcements and the polymer, suggesting that the chemical bonding of the denture base polymer to the fiber reinforcements was not adequate.^37^ Delamination consumes energy and causes a crack to deviate from the initial direction, and the energy is not used to drive cracks through the next material but to drive the deflected crack along the interface between the denture base and reline resins.^20^ It is likely that the cracks must have been propagated through the interface between the L and U materials without deflection. As a result, the IS, which is the energy needed to cause a material to fracture when struck, was higher for the L/T, L/NT, and L/K specimens than for the L/U specimens. The results observed for the L/U specimens could also be attributed to the lower IS of the autopolymerizing reline resin U compared to the heat-polymerized denture base acrylic resin L.

For the majority of the experimental conditions evaluated, thermocycling did not affect the IS of the materials. However, for the reline resin T bulk specimens, thermocycling resulted in significantly lower IS. Although one could argue that the reduced IS of T for the thermocycled group was due to the effect of water absorption alone and that thermocycling may not be a factor, it has been observed that storage in water at 37°C for up to 6 months did not affect the IS of reline resin T.^21^ In addition, Archadian et al.^5^ also observed that thermocycling led to a decrease in the flexural strength of the reline polymer-only specimens. These results suggest that for some materials, besides the plasticizing effect of the absorbed water, the increased temperature may also alter their mechanical properties. It is important to emphasize, however, that the reduction observed when resin T was tested alone was not observed when combined with resin L (L/T specimens). Given that during clinical use, the forces would be applied to the relined denture base as a whole, the results from the L/T specimens can be considered more clinically meaningful. The results of this study also showed that the L/L specimens exhibited a small but significant decrease after thermocycling. Despite this reduction, the mean value of the L/L thermocycled specimens was not significantly different from that of L intact thermocycled specimens, which in turn did not differ from that obtained for L intact nonthermocycled specimens. In addition, the statistical analysis did not show significant difference between L and L/L nonthermocycled specimens. In a previous study,^16^ it was observed that the microleakage, which can be an early indication of debonding, between hard chairside relines and denture base acrylic resins, including the combinations L/L, L/T, L/U, and L/K, was not significantly influenced by thermal cycling. Thus, it can be supposed that the decrease in the IS of L/L specimens after thermocycling would probably not significantly affect the clinical performance of the relined dentures.

The results on the strength of intact and relined denture-shaped specimens, loaded in compression until fracture, have suggested that the thermal and mechanical stresses are mechanisms that act independently and exhibit differences in the degree to which they contribute to the degradation of denture bases.^38^ Therefore, the use of a simple rectangular-shaped specimen rather than a complex denture base and the absence of cyclic loading to simulate the masticatory forces are limitations of this in vitro study and should be taken into consideration in further investigations on the IS of these materials.

## Conclusions

Within the limitations of this in vitro study, the following conclusions were drawn:

1. The mean ISs of the intact resin L specimens were significantly greater than those of the other intact materials.
2. There were no significant differences between the mean ISs of the intact L and relined L/L specimens.
3. The highest mean ISs overall were observed for L/T, L/NT, and L/K and the lowest for L/U.
4. Thermocycling did not significantly affect the IS of corresponding groups, with the exception of T and L/L for which a significant reduction was observed.
